# Twelve weeks of physical exercise breaks with coordinative exercises at the workplace increase the sulcal depth and decrease gray matter volume in brain structures related to visuomotor processes

**DOI:** 10.1007/s00429-023-02732-w

**Published:** 2023-12-09

**Authors:** Carina Scharf, Karl Koschutnig, Thomas Zussner, Andreas Fink, Markus Tilp

**Affiliations:** 1 Institute of Human Movement Science, Sport and Health, University of Graz, Mozartgasse 14, 8010 Graz, Austria; 2 Institute of Psychology, University of Graz, Universitätsplatz 2, 8010 Graz, Austria

**Keywords:** Juggling, Balance, Adult, Cortical thickness, Brain volume

## Abstract

Physical exercise can evoke changes in the brain structure. Consequently, these can lead to positive impacts on brain health. However, physical exercise studies including coordinative exercises are rare. Therefore, in this study, we investigated how 12 weeks of physical exercise breaks (PEBs) with coordinative exercises, focusing mainly on juggling tasks, affected the brain structure. The participants were randomly allocated to an intervention group (IG, *n* = 16; 42.8 ± 10.2 years) and a control group (CG, *n* = 9; 44.2 ± 12.3 years). The IG performed the PEBs with coordinative exercises twice per week for 15–20 min per session. Before the intervention, after 6 weeks of the intervention, and after 12 weeks of the intervention, participants underwent a high-resolution 3T T1-weighted magnetic resonance imagining scan. Juggling performance was assessed by measuring the time taken to perform a three-ball cascade. A surface-based analysis revealed an increase in vertex-wise cortical depth in a cluster including the inferior parietal lobe after 6 and 12 weeks of training in the IG. After 12 weeks, the IG showed a decrease in gray matter (GM) volume in a cluster primarily involving the right insula and the right operculum. The changes in the GM volume were related to improvements in juggling performance. No significant changes were found for the CG. To conclude, the present study showed that regular engagement in PEBs with coordinative exercises led to changes in brain structures strongly implicated in visuomotor processes involving hand and arm movements.

**Supplementary Information:**

The online version contains supplementary material available at 10.1007/s00429-023-02732-w.

## Introduction

Throughout the lifespan, physical exercise, which is a planned, structured, and repetitive form of physical activity (= all muscle induced bodily movements, which leads to an increased energy expenditure) (Herold et al. 2022), is positively linked with physical health, mental health (Hillman et al. 2008), and maintenance of brain health (Cabral et al. 2019). Physical exercise can also prevent the development of neurodegenerative diseases (Kramer and Erickson 2007) and cognitive decline (Ma et al. 2017). Consequently, physical activity has become an important lifestyle factor to prevent cognitive health.

The volume of the gray matter (GM) of the brain changes with age (Fjell and Walhovd 2010), but regular physical activity can prevent those age-related changes. For example, Wittfeld et al. (2020) conducted a cross-sectional study involving adults aged between 21 and 84 years. They found that cardiorespiratory fitness, developed from a regular level of physical activity, is strongly positively related to the gray matter (GM) volume of the frontal lobe, temporal lobe, the hippocampal gyrus, and the cingulate cortex. In older adults, cardiorespiratory fitness and a regular level of physical activity were positively linked to the GM volume of the prefrontal cortex and the hippocampus (Erickson et al. 2014). Furthermore, in older adults, regular level of physical activity correlates positively with the GM volume of the frontal lobe, temporal lobe of the brain, and the hippocampus (Domingos et al. 2021; Erickson et al. 2014). A positive relationship between the GM volume of the hippocampus and a regular level of physical activity was also found for young to middle-aged adults (Killgore et al. 2013). In addition, in a recent cohort study by Fox et al. (2022), an association between a regular level of physical activity with larger total GM volume was found in adults aged between 30 and 94 years.

However, most studies which have investigated the impact of a regular level of physical activity or physical exercise on brain structure and/or function have focused mostly on aerobic, anaerobic, and resistance training (Ai et al. 2021; Alkadhi 2018; Erickson et al. 2014; Gaertner et al. 2018; Herold et al. 2019; Hillman et al. 2008; Sexton et al. 2016; Smith et al. 2010; Voelcker-Rehage and Niemann 2013; Voss et al. 2011; Wilke et al. 2019; Wittfeld et al. 2020). Although the first study that showed structural brain changes due to exercise in young healthy participants was based on juggling (Draganski et al. 2004), there are fewer studies investigating the effects of coordinative training on brain structure (see Voelcker-Rehage and Niemann 2013). For example, Niemann et al. (2014a) examined how coordinative training, which included exercises to improve eye–hand and leg–arm coordination, balance, and spatial orientation and reaction, affects the brain structure in older adults. They found an increase in the GM volume of the hippocampus (Niemann et al. 2014a) and the basal ganglia (Niemann et al. 2014b). Increases in the GM volume of the hippocampus (Rehfeld et al. 2017), and the parahippocampal region (Müller et al. 2017) were found after dancing training in elderly adults. Furthermore, increases after dancing training were found in the right subiculum, the left dentate gyrus (Rehfeld et al. 2017), left precentral gyrus (Müller et al. 2017; Rehfeld et al. 2018), postcentral gyrus, left supplementary motor area, left superior temporal gyrus, medial frontal gyrus, left insula, the anterior, and medial cingulate cortex (Rehfeld et al. 2018). After balance training, increases in GM volume were found in the left supplementary motor area (SMA), superior frontal gyrus, and medial orbitofrontal cortex (Taubert et al. 2010), but decreases in GM volume were found in the putamen (Rogge et al., 2018; Taubert et al., 2010), inferior orbitofrontal cortex, middle temporal gyrus, left inferior occipital gyrus, cerebellum bilaterally (Taubert et al. 2010), right superior temporal gyrus, and left parahippocampus (Weber et al. 2019). Juggling interventions led to increases in the GM volume of the hMT + /V5 (Boyke et al. 2008; Driemeyer et al. 2008), which is an area located at the occipito-temporo-parietal pit (Sousa et al. 2016), with relevance for hand movements (Oreja-Guevara et al. 2004) and processing tactile and visual motion direction information (van Kemenade et al. 2014). Further increases were found in the frontal, temporal (Driemeyer et al. 2008), parietal lobes (Draganski et al. 2004; Driemeyer et al. 2008), the hippocampus, and the nucleus accumbens (Boyke et al. 2008). Although Sampaio-Baptista et al. (2014) found no main effect of time after 6 weeks of juggling training on the GM volume, increases in GM volume in the occipital lobe and parietal lobe, and decreases in the superior temporal gyrus, the insula, and the operculum were found during a follow-up period 4 weeks after the training.

Besides the assessment of volumetric measures, the measurement of sulcal measures can be useful. Lamont et al. (2014) indicated that changes in brain structure due to regular physical activity can be detected earlier in sulcal measures compared to volumetric measures. Of the previous published studies on coordinative exercise, three studies included measures on cortical thickness (Rogge et al. 2018; Taubert et al. 2016; Weber et al. 2019) but, to our knowledge, no one included the variables sulcal width or depth. Especially the intraparietal sulcus has been found as being strongly implicated in visuomotor and cognitive functions (e.g., Capizzi et al. 2023; Davare et al. 2012; Grefkes and Fink 2005; Richter et al. 2019). Therefore, it would be important including these measures to assess the effects of coordinative exercise, which strongly draws on visuomotor task demands.

Nevertheless, the current state of research does not provide a sufficient amount of evidence to assess the influence of physical exercise including coordinative exercises on brain outcomes in young and middle-aged adults (Erickson et al. 2019). Another challenge is to reach this age group since the largest group of adults typically spend about half of their waking hours during weekdays at the workplace (Conn et al. 2009). Therefore, implementing a physical exercise intervention in their spare time might reduce the adherence, due to multiple other distractions and time restrictions. In contrast, the workplace offers a low-threshold opportunity to reach this age group (WHO 2018). Furthermore, office workers are confronted with demanding cognitive tasks, and if they are unable to perform those tasks, it could have a negative influence on mental well-being (Bridger and Brasher 2011). Therefore, it would be helpful to integrate coordinative exercises, like juggling, at the workplace because higher level cognitive processing is required for performing coordinative exercises (Voelcker-Rehage and Niemann 2013) and, compared to other exercise types, coordinative exercises are more beneficial for improving cognitive functions (Ludyga et al. 2020). In addition, performing coordinative exercises could lead to improvements in brain areas, e.g., frontal cortical (Driemeyer et al. 2008), parietal cortical (Draganski et al. 2004; Driemeyer et al. 2008; Sampaio-Baptista et al. 2014), and hippocampal area (Boyke et al. 2008), which are related to (higher) cognitive functions (Agosta et al. 2017; Behrmann et al. 2004; Borders et al. 2022; Frith and Dolan 1996; Helfrich and Knight 2019; Lisman et al. 2017; Opitz 2014; Toichi et al. 2004). Moreover, participating in a physical exerercise break (PEB) with coordinative exercises could be useful in preventing age-related structural (Fjell and Walhovd 2010; Kochunov et al. 2005; Steffener 2021) brain changes, and brain changes due to neurodegenerative diseases (e.g., Parkinson’s and Alzheimer’s disease) (Baghdadi et al. 2022; Chishiki et al. 2020; Pereira et al. 2012; Pettigrew et al. 2017; Ramírez-Ruiz et al. 2005). Furthermore, almost all studies so far implemented coordinative exercises with a higher training duration (> 75 min per week, Sampaio-Baptista et al. 2014), and if juggling training was performed, the training mostly focused on learning the three-ball cascade (Boyke et al. 2008; Draganski et al. 2004; Driemeyer et al. 2008; Sampaio-Baptista et al. 2014). However, since insufficient time (Justine et al. 2013) to exercise and exercise-related boredom (Velasco and Jorda 2020; Wolff et al. 2021) play a role for participating in physical exercise a high training volume combined with monotonous exercise tasks might represent a threshold for average office workers to start physical exercise.

Therefore, the aim of the present study was to investigate how 12 weeks of physical exercise breaks (PEBs) with coordinative exercises with a low training volume and a variety of juggling exercises performed at the workplace affects the brain structure in young and middle-aged adults. We hypothesized that the PEBs will lead to increases in the GM volume, and the surface-based brain metrics, especially in regions related to visuomotor tasks.

## Methods

### Study participants

**Fig. 1:**
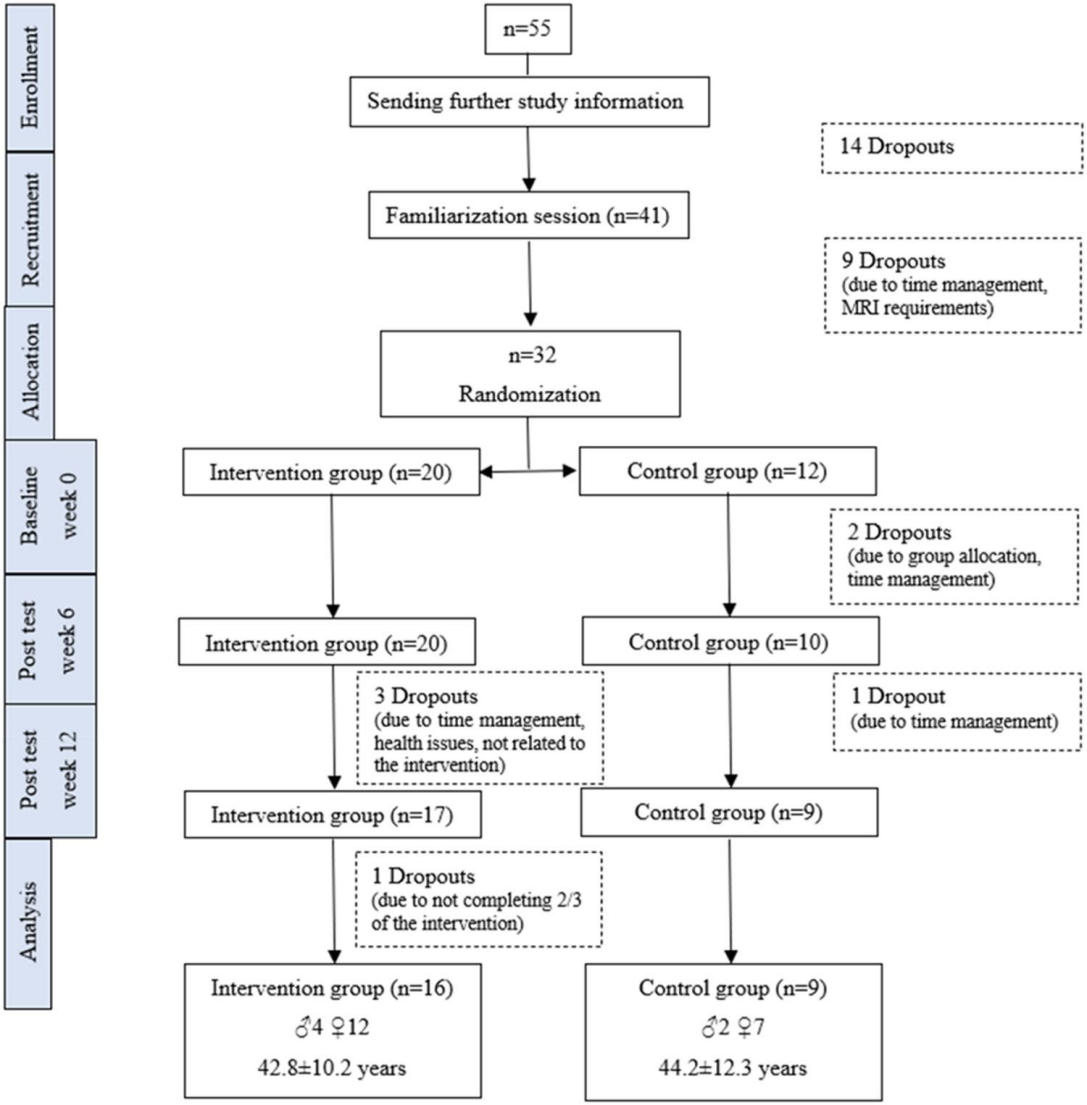
Flow diagram of participants

An exact sample size calculation based on brain structure variables was not possible because the effect of low-volume coordinative exercises has not been studied in the past. Therefore, we decided to include a convenience sample of *n* = 20 in the intervention group based on the resource constraints of the number of participants willing to participate in an MRI study. A total of 55 participants were recruited in spring and fall of 2021 via email and via advertisement on the employees’ website of the University of Graz (Austria). The inclusion criteria for the recruitment were 20–65 years, no regular engagement in intense coordinative and/or motor exercises (e.g., juggling, playing the piano), no cardiovascular, psychiatric, and/or neurological diseases, no intake of psychotropic drugs, no metallic or electrically conductive implants or prostheses in or on the body, no metal fragments in the body, no tattoos on the head and/or neck area (including cosmetic manipulations), and no pregnancy. The inclusion criteria were assessed with a standardized questionnaire, except the regular engagement in intense coordinative and/or motor exercises was assessed via self-reports. Due to losing interest in participating in the study, time management and not fulfilling at least one or more inclusion criteria, 32 participants started with the study. The participants were matched in groups of three or pairs by gender and age and were then randomly allocated to the intervention (IG) or control group (CG). The group allocation was conducted by rolling a dice. The allocation to the groups was at the rate of 2:1 (IG vs CG). The higher allocation to the IG was used to ensure that enough participants performed the PEBs. At baseline (week 0), after 6 (week 6) and 12 weeks (week 12), the participants performed the measurements, including a MRI scan and the assessment of the juggling performance. The participants performed the PEB before the measurement at least at the day before the measurement. The assessors of the MRI measurement and analyzes were blinded, but not the assessor of the juggling performance. This was due to the fact that this assessor was responsible to control if the participants performed the physical exercise breaks, what was indicated of the participants on an online platform. Since the participants logged in with their university account to the online platform, it was not possible to carry out the control of the participation for the assessor without knowing the participants names. Only participants who performed all measurements at the three measurement time points, and in case of the IG, completed at least two-thirds of the intervention sessions (16 of 24 sessions) were included in the statistical analysis. This resulted in 25 participants who were included in the final analysis (Fig. 1). All participants gave their written informed consent to participate in the study, which was in accordance with the Declaration of Helsinki and approved by the local authorized ethics committee of the University of Graz (GZ. 39/29/63 ex 2020/21). This study has not been preregistered.

### Intervention

The participants in the IG performed the PEBs, which mainly consisted of juggling tasks, for 15–20 min twice a week for 12 weeks (24 sessions). The two sessions per week were conducted on non-consecutive days. After the training program was introduced to the participants by a sport scientist, they performed the PEBs on their own via online training videos. The videos always started with a short warm-up, which included mobilization exercises (~ 1 min). The participants then practiced the juggling exercises (~ 10–15 min). The difficulty of the juggling exercises increased from simple throwing and catching tasks with one or two different objects at the beginning to juggling exercises with three objects in the last weeks. The session ended with a relaxation exercise (~ 2–3 min). Once a week, the participants performed a balance task (~ 3 min) before the relaxation part. For further details of the intervention, see Scharf and Tilp (2023).

### Demographic data and physical activity

At all three measurement sessions—baseline week 0, post-test week 6, and post-test week 12—demographic and physical activity data were collected via a self-created questionnaire. The participants were asked how many minutes per week they performed moderate physical activities, vigorous physical activities, and strengthening exercises in the last 6 weeks. Furthermore, they were also asked how much they enjoy physical activity on a scale from 1 to 4 (1 = I do not enjoy physical activity; 4 = I enjoy physical activity very much).

### Magnetic resonance imaging data acquisition

All participants underwent structural imaging on a 3T MAGNETOM Vida scanner (Siemens Healthineers, Erlangen, Germany) using a 64-channel head coil. A T1-weighted MPRAGE sequence was acquired, which took about 10 min (TR = 2530 ms, TI = 1200 ms, TE = 3.88 ms, matrix = 320 × 320, FOV = 224 mm, 192 slices, thickness 0.7 mm, no gap, no PAT, FA = 7°).

### Data quality and deidentification

Firstly, facial features were removed from all the T1-weighted images to ensure complete deidentification (pydeface, 10.5281/zenodo.3524401). Then, to test the stable imaging data quality, we opted to use MRIQc (v 0.15.0, 10.1371/journal.pone.0184661). This quality control tool enables the use of a broad spectrum of quality indices, including the signal-to-noise ratio and entropy focus criterion. A selection of the most relevant quality indices was analyzed with a mixed model and revealed a stable quality in all the relevant parameters over the time points (Table S1).

### MRI data processing and analysis

Longitudinal analyses were performed using the Computational Anatomy Toolbox (CAT r1932), implemented in MATLAB 9.6 (MathWorks, Inc., Natick, MA, USA) and Statistical Parametric Mapping (SPM12 v7771). Firstly, all the images were automatically reoriented (center of mass) and intra-subject co-registration was computed between all the images of the three time points. Next, the images were skull stripped, realigned across all subjects, and then bias corrected with regard to the mean image. This mean image was computed from all three time points for each subject separately. Next, all the images, including the mean images, were segmented with the Spatial-Adaptive Maximum A Posterior (AMAP) approach to accurately classify the three tissue types: GM, white matter, and cerebrospinal fluid. Tissue segments were then spatially normalized into the MNI (Montreal Neurological Institute) space employing DARTEL (Diffeomorphic Anatomical Registration Through Exponentiated Lie Algebra) and the geodesic shooting algorithm. Next, the modulation of the normalized tissue segments was computed for each participant based on the Jacobian determinant. This is an essential step to account for local warping and global affine transformation. Finally, the modulated data were smoothed with a 9 mm full-width at half-maximum (FWHM) smoothing kernel. In addition, the surface-based metrics were estimated using the fully automated pipeline in CAT12. The project-based thickness (PBT) approach was used for the cortical thickness and central surface estimation. The PBT approach includes topology correction, spherical mapping, and spherical registration. Parameters for the cortical thickness and folding (cortical depth) were extracted for each subject. Finally, we computed the weighted overall image quality index for all the participants and time points (mean of 2.02 ± 0.121) to ensure a sufficient data quality.

### Juggling performance

The participants performed five trials of the three-ball cascade for as long as possible, to assess their juggling performance (Boyke et al. 2008; Draganski et al. 2004; Driemeyer et al. 2008). The time (seconds) was recorded for each of the five trials, and the time from the best performance was used for the analysis.

### Statistical analysis

For the statistical analysis of the demographic, physical activity, and juggling performance data, SPSS Statistics (version 27.0; IBM, New York, USA) was used. All the analyses were performed after checking for normal distribution of the data via the Shapiro–Wilk test, and the level of significance was set to 5%. When the data were normally distributed, mixed analyses of variance (ANOVAs) with factor interaction (time × group) were used. We applied Greenhouse–Geisser correction, if the Mauchly’s sphericity test had been significant. If a significant interaction effect (time × group) was observed, post hoc tests were performed via paired t-tests within groups applying Bonferroni–Holm corrections (Holm 1979). Baseline values between groups were tested with Welch *T*-tests to account for different sample size (Delacre et al. 2017). Estimates of effect sizes are given in terms of the partial eta-squared measure (*η*_p_^2^). The effect size was determined by Cohen’s standard (≥ 0.8 = large; < 0.8 to > 0.2 = medium; ≤ 0.2 = small) (Zhu 2016). A Friedman test, Wilcoxon test, and Mann–Whitney U test were applied if the data were not normally distributed. The effect sizes for the non-parametric data were calculated with the formula $r = \frac{z }{{\sqrt {N} }}$ (Fritz et al. 2012). Due to the not normally distributed juggling performance variable, a partial Spearman’s correlation, where we accounted for age and sex, was used to analyze the relationship between the changes (over 6 and 12 weeks) in the MRI variables and the changes (over 6 and 12 weeks) in the juggling performance variable for the IG. The magnitude of the correlation (0–0.19 = no correlation; 0.2–0.39 = low correlation; 0.4–0.59 = moderate correlation; 0.6–0.79 = moderately high correlation; ≥ 0.8 = high correlation) was rated by the recommendations of Safrit and Wood (1995 as cited in Zhu 2012).

To determine intervention-related changes in GM volume, we employed a flexible factorial model with the factors group (IG, CG) and time (week 0, week 6, and week 12) implemented in SPM12. Since we were interested in time-dependent changes, only within-group changes over the different time points of assessment were computed. Therefore, we did not include age, sex, or total intracranial volume as covariates. An absolute threshold of 0.1 was applied to exclude voxels with a probability of having less than 10% GM. We then compared the changes in all three time points (week 0 vs. week 6, week 6 vs. week 12, and week 6 vs. week 12) within each group. Finally, statistical thresholds were set using a cluster-level *p*(FWE) of < 0.05, with an initial cluster forming a voxel level of *p* < 0.005. The spatial cluster size was set to 20 voxels. The same analysis strategy was applied to the surface-based analyses. We looked at changes in cortical thickness and cortical folding. The spatial extent was set to 20 vertices.

## Results

During the intervention period, four participants of the IG and three participants of the CG dropped out of the study (Fig. 1). At least 2/3 of the 24 intervention’s sessions was completed by 16 participants of the intervention group. Of those, 12 participants completed all 24 sessions (100.0%), two completed 22 sessions (91.7%), and one participant each completed 21 (87,5%), and 16 sessions (67.0%). The overall attendance adherence was 96.1% (± 8.7%). No adverse events occurred during the intervention. Accordingly, 25 participants were included in the final analysis (Fig. 1). Moreover, we excluded one participant from the CG from the analysis of the juggling performance data who was already a skilled juggler (but was not performing juggling training before or during the intervention).

### Demographic data and physical activity

A total of 25 participants (19 females, 6 males) were included in this study. Sixteen participants (12 females, 4 males) were in the IG and 9 (7 females, 2 males) served as the CG. At baseline, there were no significant differences in the variables of gender (*χ*^2^ = 0.024, *p* = 0.876), age (*F*(1,14.23) = 0.086, *p* = 0.774), BMI (F(1,18.14) = 0.103, *p* = 0.752), and enjoyment of physical activity (χ^2^ = 2.350, *p* = 0.309) (Table 1). Furthermore, no differences between the amount of moderate and high physical activity or strengthening training were found for any of the three measurement time points between the groups. In the IG, for the amount of strengthening training, and in the CG, for physical activity with high intensity, Friedman test showed a significant effect of time. However, following Bonferroni–Holm correction, there was no difference between the time points.

**Table 1:** Demographic data of the participants of the intervention group (IG) (*n* = 16) and control group (CG) (*n* = 9) at week 0

	Intervention group	Control group
Age (years) [age range]	42.8 ± 10.2 [26–61]	44.2 ± 12.3 [29–61]
Gender (n, % female)	12 (75.0%)	7 (77.8%)
BMI (m^2^/kg)	23.2 ± 2.7	22.9 ± 2.4
Enjoyment of physical activity and sports	3.8 ± 0.6	3.6 ± 5.2

The descriptive values are represented as mean ± standard deviation; [minimum–maximum]

### Gray matter (GM) volume

**Fig. 2:**
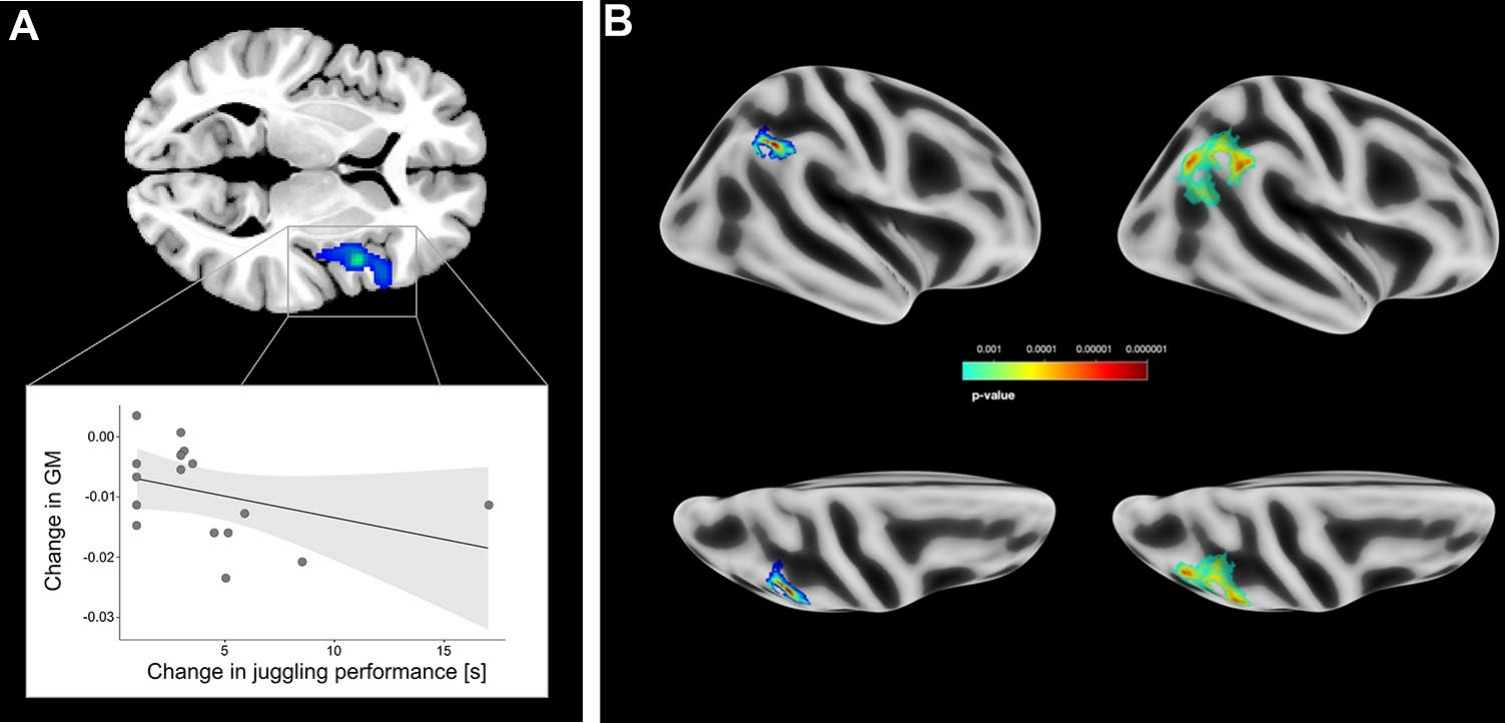
**A** Decrease in GM volume in the right Rolandic operculum/right insula after the intervention (week 12). **B** Increase in vertex-wise cortical depth in the right inferior parietal/supramarginal regions from week 0 to week 6 (left column) and from week 0 to week 12 (right column)

Analyses of GM volume revealed focal decreases in the IG after 12 weeks of training in a cluster involving the right Rolandic operculum, the right insula, and smaller portions of the right inferior frontal gyrus (X/Y/Z = 45/9/4, cluster size 2470, *p* = 4.6e–0; Fig. 2a). No effects over time were found for the CG. The Spearman’s correlation revealed a significant moderate negative relationship between changes in GM and the change in juggling performance after 12 weeks of training (*r* =  − 0.64, *p* = 0.015). Participants with higher increases in juggling performance showed stronger GM decreases in the right insula.

### Surface-based measures

In addition to intervention-related changes in GM volume, we also analyzed changes in the surface-based metrics. Applying the same statistical thresholds as for the volume-based analysis, we found significant changes in cortical depth (Table 2). More precisely, an increase in cortical depth after 6 weeks of training was observed in the right inferior parietal and supramarginal regions (Fig. 2b). In this very same region but spatially more extensive, we also found increases between the first and the third time point. No increases were found between week 6 and week 12. Mimicking the results of GM volume, no changes were found for the CG.

**Table 2:** Overview of the clusters showing significant changes in cortical depth after 6 and 12 weeks of training

	No. of vertices	*p*-value
Cortical depth/region		
Increase from week 0 to week 6		
Inferior parietal, supramarginal	118	0.00002
Increase from week 0 to week 12		
Inferior parietal, supramarginal	344	0.00002

### Juggling performance

**Fig. 3:**
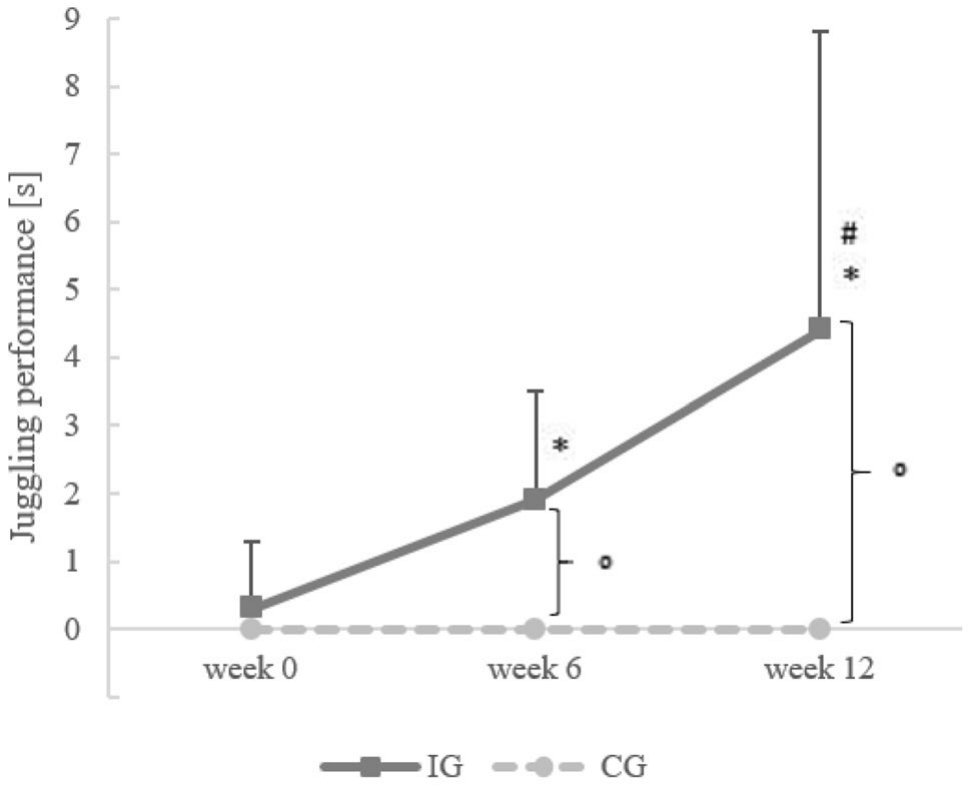
Mean and standard deviation of the three-ball cascade absolute time before (week 0), during (week 6), and after (week 12) the intervention for the IG (*n* = 16) and CG (*n* = 8; one participant was excluded due to their very high juggling skills at week 0). *Significant difference to week 0 for the IG, ^#^significant difference to week 6 for the IG; °significant difference between the IG and CG

The Mann–Whitney U test for the analysis of the juggling performance showed a significant difference between the IG and CG for week 6 (*U* = 0.00, *r* = – 0.83, *p* < 0.001) and week 12 (*U* = 0.00, *r* = – 0.82, *p* < 0.001), but not for week 0 (*U* = 60.00, *p* = 0.834) (Fig. 3). In addition, Friedman test (*χ*^2^(2) = 30.400, *p* < 0.001) showed a significant effect of time for the IG, and the Wilcoxon test analysis indicated a significant increase for the IG between week 0 and week 6 (0.3 ± 1.0 vs. 1.9 ± 1.6 s; *z* =  − 3.568, *r* = – 0.89, *p* < 0.002), week 6 and week 12 (1.9 ± 1.6 vs. 4.4 ± 4.4 s; *z* =  − 3.061, *r* = – 0.77, *p* = 0.002), and week 0 and week 12 (0.3 ± 1.0 vs. 4.4 ± 3.9 s; *z* =  − 3.534, *r* = – 0.88, *p* < 0.001). No significant change in the juggling performance was detected in the CG.

## Discussion

Regular engagement over 12 weeks in low-volume PEBs with coordinative exercises was associated with an improvement in juggling performance and an increase in vertex-wise cortical depth in a cluster including the right inferior parietal lobe. In addition, a reduction of GM volume in a cluster primarily involving the right insula and the right operculum following the intervention was also observed. Decreases in GM volume of the right insula/operculum were correlated with improvements in juggling performance. This corroborates nicely with the fact that the volumetric decreases in this brain structure were related to the juggling training.

The increase in the sulcal depth in the inferior parietal lobe following juggling training is a novel finding. Sulcal depth is a measure of cortical shape (van Essen 2005), which has been associated with neurodevelopmental disorders (van der Meer et al. 2021), working memory performance (van der Meer et al. 2021; Yao et al. 2022), and changes of the structural characteristics of the aging brain (Jin et al. 2018). In one of the rare studies on sulcal characteristics in the context of physical activity, Lamont et al. (2014) reported evidence that higher physical activity was associated with larger sulcal width, while there was no significant relationship with sulcal depth. The identified sulcal cluster in this study appears to display some overlap with areas of the intraparietal sulcus, which have been referred to as “interfaces between the perceptive and motor systems for controlling arm and eye movements in space” (Grefkes and Fink, 2005, p.3). Grefkes and Fink (2005) summarized evidence from lesion studies and brain imaging studies revealing that this brain structure is implicated in tactile and visual object processing, and especially in cross-modal information integration between the visual and the sensorimotor systems. The medial portion of the intraparietal sulcus is thought to be implicated in tasks requiring visuomotor coordination of hand movements, especially in “transforming visual coordinates into motor programs, and for the online control of goal-directed precision movements” (Grefkes and Fink, 2005, p.9). Hence, the current study provides the first evidence that structural changes of the intraparietal sulcus can constitute an important biomarker for changes following a juggling training that poses relatively high visuomotor demands.

The decrease of GM volume in response to our 12-week long juggling training was restricted to a cluster involving the right insula, the right operculum, and the right inferior frontal gyrus. The right inferior frontal gyrus is a part of the Broca’s region and represents motor functions, including complex hand movements, associative sensorimotor learning, and sensorimotor integration (Binkofski and Buccino 2004). In a quite similar vein, the insula is known to be implicated in hand–eye movements (Fink et al. 1997), proprioceptive functions (Chilvers et al. 2021), and in sensorimotor and vestibular functions (Uddin et al. 2017). The frontal operculum has been found to be specifically involved in monitoring performance during goal-directed hand movements (Quirmbach and Limanowski 2022). Interestingly, when using transcranial magnetic stimulation, Tunik et al. (2008) even demonstrated a possible causal role of the inferior frontal gyrus opercularis in planning hand–object interactions. The observed training-related GM changes in this brain structure provide empirical support for the idea that this structure is of importance for visuomotor processes being involved in the coordination of hand and eyes. The observed association between brain changes in this structure and juggling performance nicely supports this finding. However, a somewhat surprising finding is that, compared to previous juggling studies, we only found decreases of GM volume after the juggling training. In previous research, mostly, though not exclusively, increases of GM volume in different areas were found (Boyke et al. 2008; Draganski et al. 2004; Driemeyer et al. 2008; Sampaio-Baptista et al. 2014). Furthermore, when comparing the brain structure of skilled and non-skilled jugglers, Gerber et al. (2014) found a higher GM density in the hMT + /V5 area, the intraparietal sulcus, and the secondary visual cortex. In particular, the GM density of the hMT + /V5 area was positively related to the performance of the skilled jugglers. However, compared to the juggling skills of the participants in the previous juggling studies, the participants of the IG of the present study were not very skilled jugglers after completing the intervention. The participants were only able to juggle for 4.4 s (SD = 4.4) after 12 weeks of training. In the other studies, participants reached or had to reach juggling times between 20 and 180 s (Boyke et al. 2008; Draganski et al. 2004; Driemeyer et al. 2008; Sampaio-Baptista et al. 2014). Nevertheless, Sampaio-Baptista et al. (2014) also found some decreases of GM volume following juggling, especially in the left superior temporal gyrus, the insula, and operculum. Moreover, in other studies where training with high coordinative demands was included, decreases of the GM volume were also found in different areas of the brain (Rogge et al. 2018; Taubert et al. 2010; Weber et al. 2019). For example, Weber et al. (2019) found that learning to ride a unicycle over a time period of 3 weeks was associated with significant reductions of GM volume in regions supporting visuospatial processes. Similarly, in professional or skilled athletes who are regularly engaged in tasks requiring a high level of coordinative skills, both greater and smaller GM volumes have been reported. Skilled golf players have a higher GM volume for the premotor cortex and parietal areas, compared to less-skilled golf players (Jäncke et al. 2009). Professional ballet dancers have a lower GM volume in the left premotor cortex, SMA, putamen, and superior frontal gyrus, compared to non-skilled dancers (Hänggi et al. 2010). The results of a cross-over study of skilled dancers (ballet and figure skating) and slackliners showed a lower GM volume in the anterior hippocampal formation and parieto-insular vestibular cortex, but a higher GM volume in the posterior hippocampal formation, lingual, and fusiform gyri, compared to a non-trained CG (Hüfner et al. 2011). Taken together, these findings clearly indicate that these decreases in GM volume do not indicate that a brain structure is “deactivated” following the training, but they may rather reflect the reorganization of brain tissue facilitating more automated and efficient task performance (in this case, visuomotor coordination; see Weber et al. 2019).

It is evident that brain changes following physical exercise vary as a function of the practice level. For example, Sampaio-Baptista et al. (2014, 2015) found that a juggling training with a lower duration of the exercise sessions (75 min per week) showed a negative correlation between the brain changes of GM volume of the left motor cortex and dorsolateral prefrontal cortex (DLPFC) with the changes in juggling performance. In contrast, a juggling training with higher duration of the exercise sessions (150 min per week), the same relationship was positive. For the white matter (WM) volume, the relationship was the opposite, i.e., a juggling training with a lower duration of the exercise sessions had increased functional connectivity and a juggling training with higher duration of the exercise sessions had decreased functional connectivity, as indicated by increased and decreased motor resting state structure strength, respectively. It is important to mention that the juggling performance was not significantly different between both these groups after finishing the juggling intervention. Weber et al. (2019) found similar effects after 3 weeks of unicycle training. They reported a decrease in the GM volume and an increase of the WM fractional anisotropy after the training. Both research groups, i.e., Sampaio-Baptista et al. (2014, 2015) and Weber et al. (2019), suggested that the changes rely on the different reorganization processes of brain tissue and are moderated by the participant’s skill or amount of practice. Weber et al. (2019) showed that the GM decreases in task-relevant structures following unicycle training subsequently increased again after a follow-up period during which no unicycle training was performed. This strongly supports the view that brain changes following physical activity are highly dynamic and strongly related to the amount of physical exercise.

This study also has some limitations. Firstly, we did not assess changes in diffusion-weighted MRI and functional characteristics of the brain following the intervention. Recent research has indicated that the combined consideration of these different imaging modalities provides a comprehensive and more holistic view of the manifold brain changes associated with coordinative exercises (e.g., Weber et al. 2019). A further limitation is the higher number of women (*n* = 19) compared to men (*n* = 6) participating in the current study. According to the literature, it is more likely that women participate in worksite programs (Beck et al. 2016). Moreover, these results are in line with other studies implementing physical exercise programs at the workplace, where a higher percentage of women (between 64.2 and 90.0%) participated in the programs (Dalager et al. 2017; Gram et al. 2014; Grande et al. 2015; Hartfiel et al. 2011; Hunter et al. 2018; Puig-Ribera et al. 2008). Finally, no a priori sample size calculation was done and the sample size of this study is small, which certainly limits the statistical power. This limitation of the statistical power could lead to a reduction of the likelihood that significant results reflect a true effect (Button et al. 2013). Nevertheless, the fact that we found brain changes in regions strongly implicated in visuomotor processes indicated that the findings are meaningful. This study was hypothesis generating and should serve as basis for future studies in this field where we await replication of these findings in larger samples of participants.

## Conclusions

The present study provides the first evidence that 12 weeks of PEBs with coordinative exercises and with a low training volume and a variety of juggling exercises performed at the workplace result in changes in GM volume and sulcal depth. These changes were found in brain structures strongly implicated in visuo-coordinative processes involving hand and arm movements. Future research in this field would benefit by the use of different brain imaging modalities. In particular, the assessment of magnetic resonance spectroscopy in the context of physical exercise could also provide important new insights into the biochemical and cellular mechanisms underlying the observed changes in brain volume.

### Supplementary Information

Below is the link to the electronic supplementary material.

Supplementary file1 (JPG 41 KB)

## Data Availability

The data presented in this study are available on request from the corresponding author. The data are not publicly available, for privacy reasons.
