# Supplementary figures and images for: Twelve weeks of physical exercise breaks with coordinative exercises at the workplace increase the sulcal depth and decrease gray matter volume in brain structures related to visuomotor processes
Source: Brain Struct Funct. 2023 Dec 9;229(1):63–74. doi: 10.1007/s00429-023-02732-w (PMC10827861; doi:10.1007/s00429-023-02732-w)

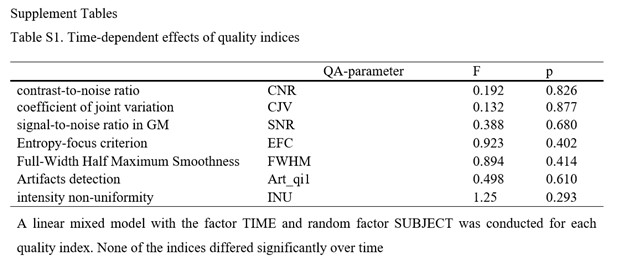

Supplement: Supplementary file 1 — Supplementary file1 (JPG 41 KB) [file 429_2023_2732_MOESM1_ESM.jpg]
